# Localization of BRCA1 protein in breast cancer tissue and cell lines with mutations

**DOI:** 10.1186/1475-2867-13-70

**Published:** 2013-07-15

**Authors:** Natalie Tulchin, Leonard Ornstein, Steven Dikman, James Strauchen, Shabnam Jaffer, Chandandeep Nagi, Ira Bleiweiss, Ruth Kornreich, Lisa Edelmann, Karen Brown, Carol Bodian, Venugopalan D Nair, Monique Chambon, Nicholas T Woods, Alvaro NA Monteiro

**Affiliations:** 1 Department of Pathology, Mount Sinai School of Medicine, 1 Gustave L. Levy Place, New York, NY 10029, USA; 2 Department of Genetics, Mount Sinai School of Medicine, New York, NY, USA; 3 Department of Anesthesiology, Mount Sinai School of Medicine, New York, NY, USA; 4 Department of Neurology, Mount Sinai School of Medicine, New York, NY, USA; 5 Faculté de Pharmacie, IBMM, Montpellier, France; 6 Cancer Epidemiology Program, H. Lee Moffitt Cancer Center and Research Institute, Tampa, FL, USA; 7 Department of Oncological Sciences, Morsani College of Medicine, University of South Florida, Tampa, FL, USA

**Keywords:** Breast cancer, *BRCA1* mutations, Frozen section immunohistology, Nucleolar localization

## Abstract

**Background:**

The breast and ovarian cancer susceptibility gene (*BRCA1*) encodes a tumor suppressor. The BRCA1 protein is found primarily in cell nuclei and plays an important role in the DNA damage response and transcriptional regulation. Deficiencies in DNA repair capabilities have been associated with higher histopathological grade and worse prognosis in breast cancer.

**Methods:**

In order to investigate the subcellular distribution of BRCA1 in tumor tissue we randomly selected 22 breast carcinomas and tested BRCA1 protein localization in frozen and contiguous formalin-fixed, paraffin embedded (FFPE) tissue, using pressure cooker antigen-retrieval and the MS110 antibody staining. To assess the impact of *BRCA1* germline mutations on protein localization, we retrospectively tested 16 of the tumor specimens to determine whether they contained the common Ashkenazi Jewish founder mutations in *BRCA1* (185delAG, 5382insC), and *BRCA2* (6174delT). We also compared co-localization of BRCA1 and nucleolin in MCF7 cells (wild type) and a mutant *BRCA1* cell line, HCC1937 (5382insC).

**Results:**

In FFPE tissue, with MS110 antibody staining, we frequently found reduced BRCA1 nuclear staining in breast tumor tissue compared to normal tissue, and less BRCA1 staining with higher histological grade in the tumors. However, in the frozen sections, BRCA1 antibody staining showed punctate, intra-nuclear granules in varying numbers of tumor, lactating, and normal cells. Two mutation carriers were identified and were confirmed by gene sequencing. We have also compared co-localization of BRCA1 and nucleolin in MCF7 cells (wild type) and a mutant *BRCA1* cell line, HCC1937 (5382insC) and found altered sub-nuclear and nucleolar localization patterns consistent with a functional impact of the mutation on protein localization.

**Conclusions:**

The data presented here support a role for BRCA1 in the pathogenesis of sporadic and inherited breast cancers. The use of well-characterized reagents may lead to further insights into the function of BRCA1 and possibly the further development of targeted therapeutics.

## Background

Germline mutations in the breast cancer tumor suppressor genes *BRCA1*[1-3] and *BRCA2*[4,5] have been found in familial breast and ovarian cancer. Prevalence of mutations in patients aged 20 to 74 years with breast cancer were reported to be 3.3%; a finding which did not provide support for screening of the general population [6]. Breast cancer patients aged 35 years or younger and individuals less than age 45 with first-degree family history of breast cancer had mutation prevalence of about 6.5 and 7%, respectively [7]. Lidereau et al. [8] found they could improve the likelihood of detecting *BRCA1* mutations in a series of patients with invasive breast cancer, under 35 years of age, when they selected patients with estrogen receptor (ER) negative, high grade tumors (37%). Approximately 2% of Ashkenazi Jews carry mutations in *BRCA1* or *BRCA2* that confer, at age 70, an estimated risk of breast cancer of 56% [9,10]. However, somatic *BRCA1* mutations have not been found in sporadic breast cancer tumor tissue [3,6] although as a tumor suppressor it is thought that *BRCA1* participates in tumorigenesis through reduction in BRCA1 mRNA, protein levels, and changes in *BRCA1* promoter methylation [11-17]. Recently, *BRCA1* mutations have been shown to render breast cancer tumors sensitive to poly (ADP-ribose) polymerase (PARP) inhibition [18]. Sensitivity to PARP inhibitors is thought to be due to a synthetic lethal combination of the inhibitor-induced single-strand break repair deficiency along with loss of the homologous recombination (HR) function of *BRCA1*[19].

BRCA1 protein immunohistological staining with the MS110 antibody has shown a speckled nuclear pattern in many breast and ovarian cancer cell lines, as well as in centrosomes and mitochondria [20,21]. Scully et al. [22] described nuclear staining in breast and ovarian cancer cell lines with four monoclonal anti-BRCA1 antibodies and after microwave heating of neutral buffered FFPE sections of primary invasive ductal carcinomas. However, Chen et al. [23] described anti-BRCA1 antibodies which detected cytoplasmic staining in a variety of normal and cancer cell lines, and mixed cytoplasmic and nuclear staining in FFPE breast cancer tissues. Wilson et al. [24] tested 19 anti-BRCA1 antibodies which detected a 220 kD protein localized to nuclear foci in epithelial cell lines from breast malignancies. In FFPE specimens, more uniform nuclear staining was observed in benign breast, invasive lobular cancers, and low grade ductal cancer, while less or absent staining was found in the majority of high-grade ductal carcinomas and breast cancer tissue from patients with *BRCA1* 185 del AG mutations. However, BRCA1 protein staining remains controversial because of questions about the specificity of antibodies, variations in staining protocols, and staining of lymphocytes in surrounding stromal tissue [25].

Our previous studies have shown that BRCA1 protein is localized in tumor cell nuclei and nucleoli in frozen tissue sections, and is co-localized with nucleolin in MCF7 and HeLa cells [26,27]. Chambon et al. [28] have shown in light and electron microscopic studies of estradiol-treated MCF7 cells that BRCA1 nuclear staining was found in dots around nucleoli, and in the cytoplasm in multivesicular bodies near the Golgi.

In the present study, we show similarities and concordance of BRCA1 protein localization between frozen and pressure cooker antigen-retrieved FFPE tissue in 22 randomly selected breast carcinomas. We further characterized our randomly selected population, by retrospectively genotyping 16 of our anonymized samples for Ashkenazi Jewish mutations *BRCA1* (185delAG, 5382insC) and *BRCA2* (6174delT). We found two specimens with mutations, one from patient no. 4, with a *BRCA2* (6174delT) mutation, and one from patient no. 13, with a *BRCA1* (185delAG) mutation, which were subsequently confirmed by DNA sequencing. With immunofluorescence staining and confocal microscopy, we were able to detect BRCA1 sub-nuclear localization in frozen breast cancer tissue specimens and co-localization of BRCA1 and nucleolin in MCF7 (hemizygous for *BRCA1* wild type), and HCC1937 (*BRCA1* 5382insC) cells.

## Results

**Figure 1:**
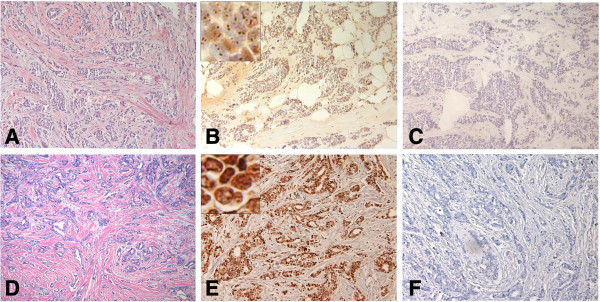
**BRCA1 protein in frozen and FFPE sections of moderately differentiated adenocarcinoma (MDA) from patient no. 2. A**. H&E stained frozen section of the tumor. **B**. Consecutive section of the same tumor stained with the mouse monoclonal AP16 antibody and the avidin-biotin peroxidase detection system showed that many of the cells exhibited multifocal dot-like nuclear staining of category (2); insert shows subnuclear foci. **C**. Negative control section using standard dilutions of mouse IgG showed essentially no staining. **D**. H&E section of FFPE tissue from patient no. 2. **E**. Consecutive section of the same tumor treated with pressure cooker antigen retrieval and stained with the MS110 antibody showed that most of the cells exhibited moderate nuclear staining of category (3); insert shows dark staining. **F**. Consecutive control section of FFPE tissue without primary antibody. (×200) inserts (×2000).

**Figure 2:**
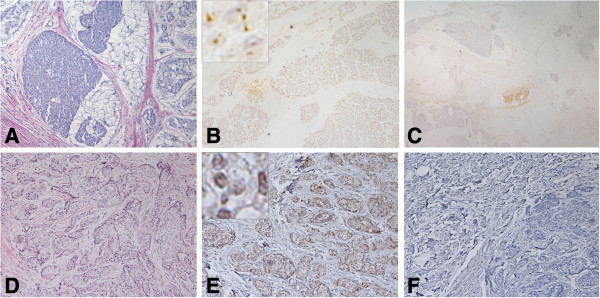
**BRCA1 protein in frozen and FFPE sections of moderately differentiated, lobular carcinoma (MDA-lob) from patient no.7. A**. H&E stained frozen section of tumor. **B**. Consecutive section of the same tumor stained with mouse monoclonal AP16 antibody and biotinylated anti-mouse IgG and avidin-biotin-peroxidase detection system showed that many of the cells showed distinct, punctate nuclear staining of category (2); insert shows focal staining . **C**. Standard dilution of mouse IgG served as a negative control. **D**. H&E section of FFPE tissue from patient no.7. **E**. Consecutive section of the same tumor treated with pressure cooker antigen retrieval and stained with the MS110 antibody showed that many of the cells exhibited distinct nuclear staining (2); insert shows light staining. **F**. Consecutive control section of FFPE tissue without primary antibody. (×200) insert (×2000).

**Figure 3:**
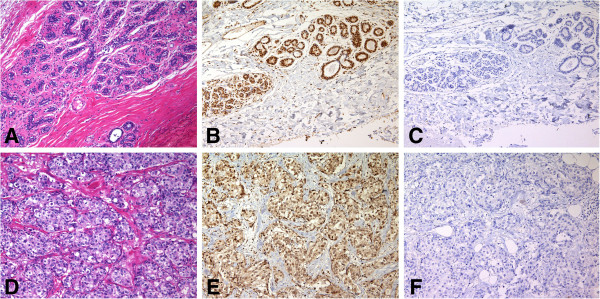
**BRCA1 protein localization in normal and tumor FFPE sections of poorly differentiated adenocarcinoma (PDA) from patient no.19. A**. H&E section of FFPE normal tissue. **B**. Consecutive section of the normal tissue stained with the MS110 antibody showed strong epithelial cell nuclear staining. **C**. Consecutive control section of FFPE tissue without primary antibody. **D**. H&E section of FFPE tumor tissue. **E**. Consecutive section of the same tumor treated with pressure cooker antigen retrieval and stained with the MS110 antibody showed that many of the cells exhibit distinct nuclear staining (2). **F**. Consecutive control section of FFPE tissue without primary antibody. (×200).

**Figure 4:**
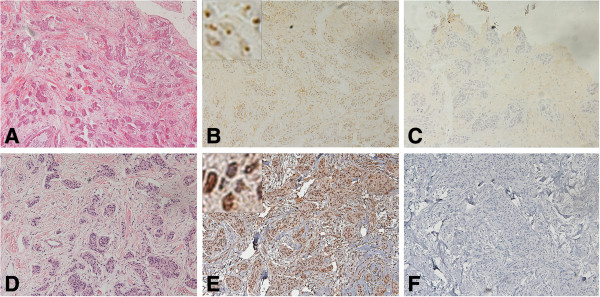
**BRCA1 protein in frozen and FFPE sections of poorly differentiated adenocarcinoma (PDA) from patient no. 4 with a *BRCA2 *(6174delT) mutation. A**. H&E stained frozen section of the tumor. **B**. Consecutive section of the same tumor stained with the mouse monoclonal AP16 antibody and the avidin-biotin peroxidase detection system showed that many of the cells exhibited moderate punctate nuclear staining (3); insert shows focal staining. **C**. Negative control section using standard dilutions of mouse IgG. **D**. H&E section of FFPE tissue from patient no.4. **E**. Consecutive section of the same tumor treated with pressure cooker antigen retrieval and stained with the MS110 antibody showed that most of the cells exhibited strong nuclear staining (4), and insert shows strong staining. **F**. Consecutive control section of FFPE tissue without primary antibody. (×200) insert (×2000).

**Figure 5:**
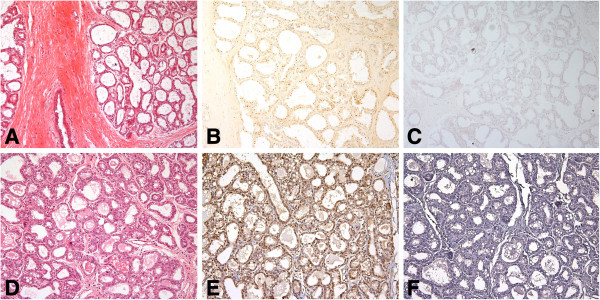
**BRCA1 protein in frozen and FFPE sections from lactating tissue from patient no**.**4 with a BRCA2 mutation. A**. H&E stained frozen section of lactating tissue. **B**. Consecutive section of the same tissue stained with the mouse monoclonal AP16 antibody and the avidin-biotin peroxidase detection system showed that many of the lactating cells showed punctate nuclear granules. **C**. Negative control section using standard dilutions of mouse IgG. **D**. H&E section of FFPE lactating tissue. **E**. Consecutive section of the same tissue treated with pressure cooker antigen retrieval and stained with the MS110 antibody showed that most of the lactating cells exhibited moderate homogeneous nuclear staining (3). **F**. Consecutive control section of FFPE tissue without primary antibody. (×200).

**Figure 6:**
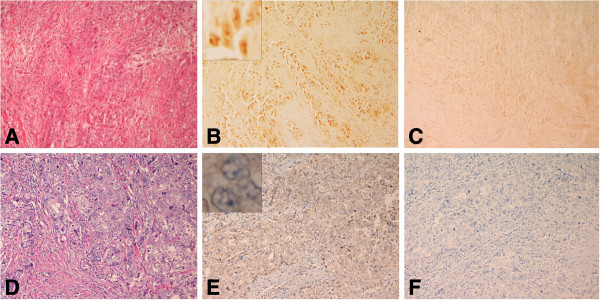
**BRCA1 protein in frozen and FFPE sections of poorly differentiated adenocarcinoma (PDA) from patient no.13 with a *BRCA1 *mutation (185delAG). A**. H&E stained frozen section of the tumor. **B**. Consecutive section of the same tumor stained with rabbit polyclonal K-18 antibody and anti-rabbit IgG and avidin-biotin peroxidase detection system. Many of the cells exhibited diffuse nuclear staining (2), and insert shows diffuse staining. **C**. Consecutive section of the same tumor incubated with control rabbit IgG shows no appreciable staining. **D**. H&E section of FFPE tissue from patient no.13. **E**. Consecutive section of the same tumor treated with pressure cooker antigen retrieval and stained with the MS110 antibody showed that many of the cells exhibited weak nuclear staining (1), and insert shows unstained nuclei. **F**. Consecutive control section of FFPE tissue without primary antibody. (×200) insert (×2000).

**Figure 7:**
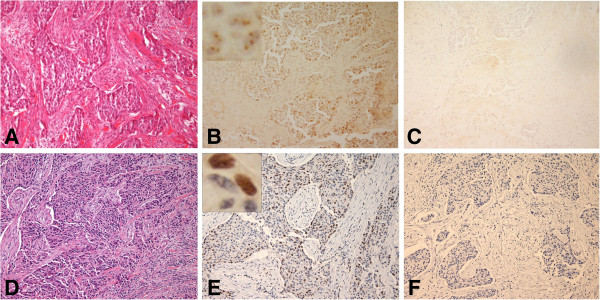
**BRCA1 protein localization in frozen and FFPE sections of poorly differentiated adenocarcinoma (PDA) from patient no. 22 using avidin**-**biotin**-**detection system. A**. H&E stained frozen section of tumor. **B**. Consecutive section of the tumor stained with mouse monoclonal AP16 antibody and biotinylated anti- mouse IgG showed that few of the cells showed distinct, punctate nuclear staining (2), and insert shows focal staining. **C**. Standard dilution of mouse IgG served as negative control. **D**. H&E section of FFPE tissue from patient no.22. **E**. Consecutive section of the same tumor treated with pressure cooker antigen retrieval and stained with the MS110 antibody showed that few of the cells exhibited distinct nuclear staining (2) and insert shows darkly stained and unstained nuclei. **F**. Consecutive control section of FFPE tissue without primary antibody. (×200) insert (×2000).

We found variable BRCA1 protein immunostaining in tumor cell nuclei in frozen tissue sections with the AP16 and K-18 antibodies and with the MS110 antibody in contiguous FFPE tissues of randomly selected breast carcinomas (Table 1, Figures 1, 2, 3, 4, 5, 6 and 7). In frozen sections, we found variable punctate nuclear staining (Figures 1, 2, 6, and 7) and we show distinct nuclear staining, and moderate staining in Figure 4. There was concordance between frozen and FFPE section staining in tumor cell nuclei amongst the histological types, with less staining with higher histopathological grade (Table 1). In FFPE stained tumor tissue, the majority of the poorly differentiated adenocarcinomas (PDAs) were in the weak, faint, (1), or distinct (2) staining categories, and less in the moderate (3), and strong (4) staining group. The MDA-PDA, and MDA, lobular specimens showed more elevated levels of staining in both frozen and FFPE sections (Table 1).

**Table 1:** Tumor genotype and BRCA1 staining

**#Specimen**	**Histology**	**BRCA1**		**ER/PR**	**Genotype**
		**FS**	**FFPE**		
1.	PDA	+++	N.A.	−/+	N.T.M.
2.	MDA	++	+++	+/+	N.T.M.
3.	PDA	+++	++	+/+	N.T.M.
4.	PDA	+++	++++	−/−	BRCA2
					6174delT
5.	PDA	++++	+++	+/+	N.T.M.
6.	MDA	++	++	+/+	N.T.M.
7.	MDA,lob	++	++	+/+	N.T.M.
8.	PDA	++	+++	+/−	N.A.
9.	Medullary	++	N.A.	−/−	N.A.
10.	Mixed,lob& duct.	++++	++++	+/−	N.T.M.
11.	PDA	++	+	−/−	N.T.M.
12.	MDA-PDA	++	++	+/+	N.T.M.
13.	PDA	++	+	−/−	BRCA1
					185delAG
14.	MDA-PDA	+++	+++	+/+	N.T.M.
15.	PDA	++	+	+/+	N.T.M.
16.	Lobular	+++	+++	+/+	N.A.
17.	PDA	++	++	+/−	N.A.
18.	MDA	+++	+++	+/+	N.A.
19.	MDA	++	++	+/−	N.A.
20.	MDA-PDA	++	++++	+/+	N.T.M.
21.	PDA	++	++	−/−	N.T.M.
22.	PDA	++	++	+/+	N.T.M.

ER/PR= Estrogen/Progesterone Receptor.

MDA= Moderately differentiated adenocarcinoma.

PDA= Poorly differentiated adenocarcinoma.

N.A.= not available.

N.T.M.= no targeted mutations.

In some of our samples, we found less positive BRCA1 nuclear staining in FFPE breast tumor tissue compared to normal tissue as in this example from patient no.19 (Figure 3).We also found strongly stained normal tissue in specimens nos. 2, 15, 20, and 22; however, we found weakly stained normal tissue from patients nos. 13, and 17.

In our 16 anonymized frozen tissue samples tested for *BRCA* Ashkenazi founder mutations, we detected two mutation carriers: patient no. 4 who was a mutation carrier for *BRCA2* (6174delT), and patient no. 13 who was a mutation carrier for *BRCA1* (185delAG). No targeted mutations were found in the other 14 samples (1,2,3,5,6,7,10,11,12,14,15,20,21,and 22). Six samples were not available (8,9,16,17,18, and 19).We assayed BRCA1 immunohistology in our specimens, including those from patients no. 4 and 13 before testing their mutation status. In the frozen sections from patient no. 4, there was moderate (3) BRCA1 nuclear staining in the tumor cells, as well as in the surrounding lactating breast tissue (Figure 5). The contiguous FFPE tissue from patient no. 4 expressed strong (4) BRCA1 nuclear staining in the tumor and in the lactating ducts (Figures 4 and 5).

**Figure 8:**
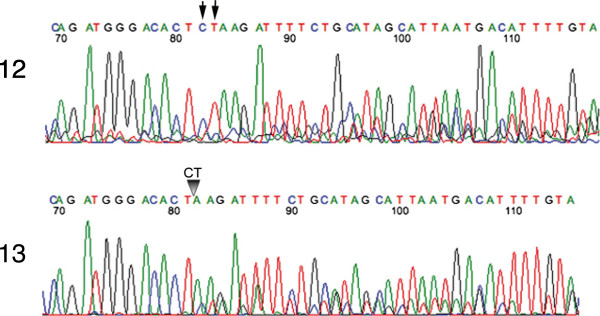
**DNA sequencing chromatogram illustrates the mutation, 185delAG, resulting from the deletion of two consecutive nucleotides AG at position 185 (**∇**) in BRCA1 gene in sample no. 13 (lower panel).** DNA sequences from sample no. 12 (wild type) are shown in top panel. Arrows indicate the position of AG deletion. In both, DNA from FFPE tissues were amplified and nucleotide sequences were determined using the primers flanking the mutation loci.

In contrast, we found diffuse, and irregular but distinct (2) BRCA1 protein staining with the K-18 antibody, in frozen tumor tissue from patient no. 13 with the *BRCA1* mutation (Figure 6) The contiguous FFPE tissue stained with the MS110 antibody was only very weakly stained (1) (Figure 6). In the chromatogram (Figure 8), the *BRCA1* 185delAG mutation in the contiguous paraffin sample for patient no. 13, is compared to the normal sequence in patient no. 12. Neither of the tumors from patient no. 4 or from patient no. 13 showed loss of heterozygosity (LOH) by homozygosity of the mutant allele, as determined by PCR of the amplified fragments (data not shown).

After assessing BRCA1 protein staining by histopathological grade, we further grouped our BRCA1 protein staining results by ER/PR status (Table 1). PDA samples comprised eight out of ten specimens that were either ER or PR negative, or were both ER/PR negative, although the data are too few to permit statistical significance. In addition, two of the ER/PR negative PDAs were mutation carriers (Table 1). It is also interesting to note that two PDA samples with moderate or strong BRCA1 staining are ER/PR positive.

**Figure 9:**
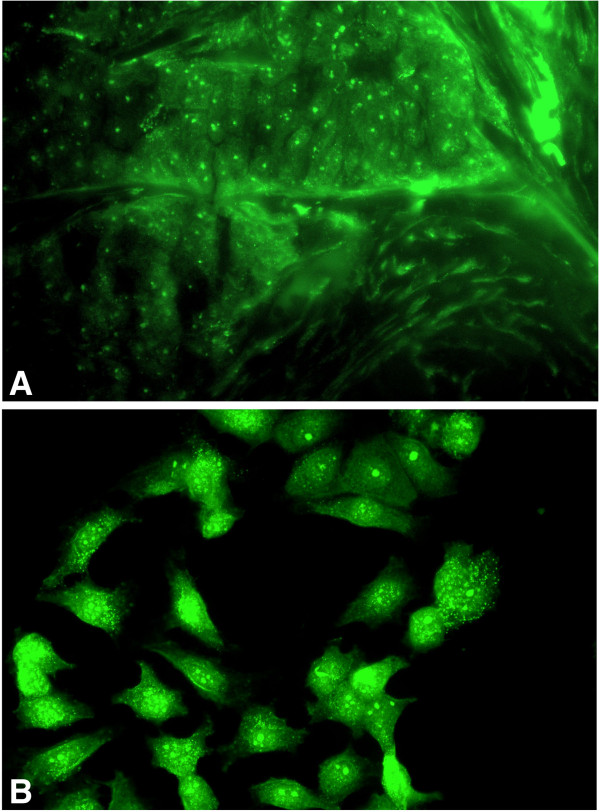
**Immunofluorescence staining of BRCA1 protein localization in poorly differentiated adenocarcinoma (PDA) from patient no.22 and an asynchronous culture of MCF7 cells. A**. The tumor is stained with rabbit polyclonal K-18 antibody and FITC-labelled goat anti-rabbit serum. Many tumor cell nuclei show both 1–5 punctate bodies (green) and large round central structures (green) and the interstitial cell nuclei are very weakly stained. **B**. Asynchronous culture of MCF7 cells stained with rabbit polyclonal K-18 antibody and FITC-labelled goat anti-rabbit. BRCA1 staining consisting of fluorescent nuclei, intra-nuclear punctate bodies (green) and cytoplasmic staining. (×300).

We compared immunoperoxidase and immunofluorescence detection procedures to study BRCA1 protein staining in the frozen and FFPE PDA tissue from patient no. 22. With the AP16 antibody and immunoperoxidase detection, many of the tumor cell nuclei showed punctate granular staining of category (2) in the frozen sections (Figure 7). Distinct nuclear staining (2) of many of the tumor cells was found in the contiguous FFPE sections with the MS110 antibody (Figure 7). We then compared immunofluorescence staining with the K-18 antibody on the frozen sections from patient no. 22; BRCA1 protein nuclear localization appeared similar, although there was greater sensitivity with immunofluorescence (Figure 9A). Between 1 and 5 punctate bodies (green) were visible in tumor cell nuclei; the interstitial cell nuclei were only very weakly stained or unstained, although there was intrinsic fluorescence in the stromal tissue. In asynchronously growing MCF7 cells, we found varying staining patterns with the K-18 BRCA1 antibody (Figure 9B). The patterns included strongly fluorescent nuclei, cells with intra-nuclear punctate bodies, and cells with nuclear and cytoplasmic staining.

**Figure 10:**
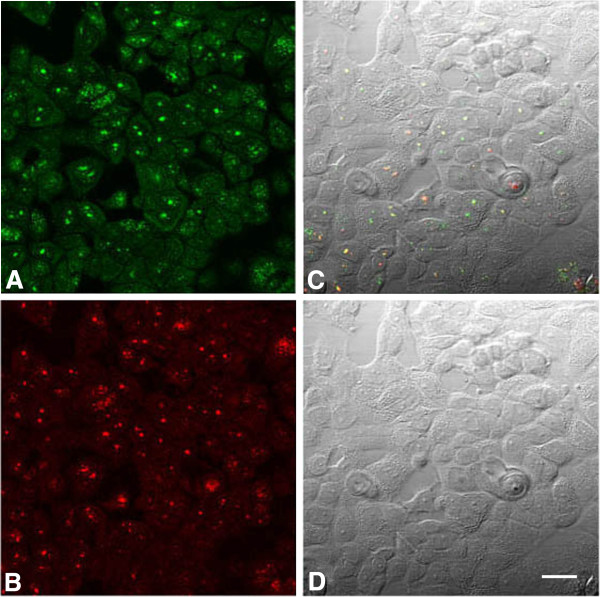
**Co**-**localization of BRCA1 antibody (K-18) and nucleolin (7G2) in MCF7 cells. A**. Rabbit polyclonal BRCA1 antibody stains many cell nuclei (green). The varied nuclear patterns include many small dot-like speckles, punctate bodies and speckles, and round centrally placed bodies. **B**. Mouse monoclonal antibody to nucleolin stains nucleoli and many dot-like speckles (red). **C**. Nuclei which stain for BRCA1 (green) merge with nucleolin (red) to show co-localization(yellow). The merge occurs both in nucleoli (which are phase dense in D) and in some nuclear speckles. Not all of the nucleoli show co-localization. **D**. Nomarski image of the same field as A,B,C. Bar = 20 μm.

**Figure 11:**
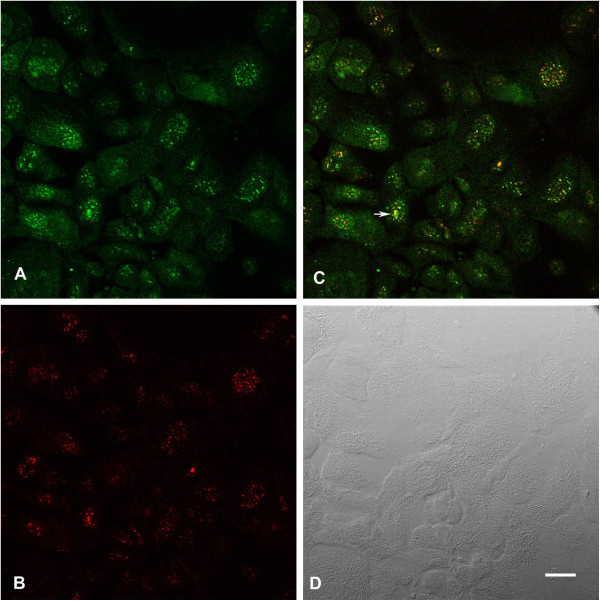
**Co**-**localization of BRCA1 antibody (K-18) and nucleolin (7G2) in HCC1937 cells. A**. rabbit polyclonal BRCA1 antibody stains many cell nuclei (green). The varied nuclear patterns include dot-like speckles, and intra-nuclear granules. **B**. Mouse monoclonal antibody to nucleolin stains nucleoli and many dot-like speckles (red). **C**. Some nuclear speckles, nucleoli, and portions of nucleoli stain for BRCA1(green) and nucleolin (red) to show co-localization (yellow). The arrow points to a nucleolus, in which there are BRCA1 (green), nucleolin (red), and merged (yellow) regions, which are distinct. **D**. Nomarski image of the same field as A,B,C. Bar = 20 μm.

We compared BRCA1 and nucleolin co-localization in asynchronously growing MCF7 cells (Figure 10) and HCC1937 cells (Figure 11). The MCF7 cells exhibited varying staining patterns with both antibodies; with the BRCA1 antibody, the nuclear patterns included nuclei with many small dot-like speckles, nuclei with punctate bodies and speckles, and nuclei with round centrally placed bodies (Figure 10A). On the same field, the mouse monoclonal antibody to nucleolin stained nucleoli and many dot-like nuclear speckles (Figure 10B). The co-localization of BRCA1 protein and nucleolin occurred frequently in nucleoli (which are phase dense in Figure 10D) and in some nuclear speckles (Figure 10C). In HCC1937 cells, co-localization of BRCA1 antibody (K-18) and nucleolin 7G2 was found in nuclear speckles, nucleoli, and portions of nucleoli, however, some of the nucleoli, exhibited patches of BRCA1 (green), nucleolin (red), and merged (yellow) regions (Figure 11).

**Figure 12:**
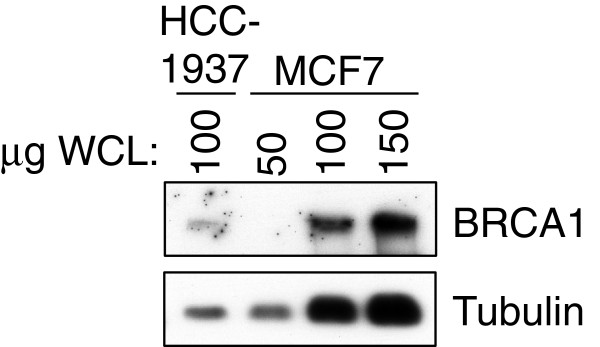
**BRCA1 protein levels in MCF7 and HCC1937 cells.** Extracts of HCC1937 and MCF7 cells were normalized for protein content, separated by 5% SDS PAGE, and immunoblotted for BRCA1 with mAb MS110. HCC1937 extracts revealed low levels of a 210 kDa BRCA1 species, when we compared 100 μg of total protein in the HCC1937 extracts (5382insC product) with 50 μg, 100 μg, and 150 μg of control MCF7 extracts (wild-type BRCA1).

We performed Western blotting to confirm BRCA1 protein levels in MCF7 and HCC1937 cells (Figure 12). Relatively low levels of a 210 kDa BRCA1 species were found in the HCC1937 extracts (5382insC product), compared to the higher levels in control MCF7 extracts (wild-type BRCA1).

## Discussion

We found concordant BRCA1 protein staining in frozen and FFPE tissue specimens of 22 randomly selected breast tumors. Using anti-BRCA1 antibodies AP16 and K-18 on frozen sections we found punctate BRCA1 staining and more homogeneous staining with the MS110 antibody in variable numbers of tumor cell nuclei and nucleoli of breast cancer patients [26,27]. There was variable homogeneous nuclear staining with the MS110 antibody in contiguous pressure cooker antigen-retrieved FFPE tumor tissue. These results extend our previous studies which showed BRCA1 nuclear and nucleolar localization in frozen tissue sections and estradiol-treated MCF7 cells [26-28]. However, Bogdani et al. [29] using D-20, K-18, I-20, and C-20 anti-BRCA1 antibodies on Bouin’s fixed tumor tissue of young and old patients with breast cancer, found nuclear and cytoplasmic BRCA1 staining in about 50% of sporadic specimens, and less staining in tumor cells in young patients and one with a germline mutation. Perez-Valles et al. [30] showed predominantly cytoplasmic staining with the D-20, I-20, and K-18 polyclonal antibodies in FFPE samples of both tumoral and non-tumoral cells, but nuclear and cytoplasmic staining with the I-20 antibody in FFPE samples after microwave pre-treatment in breast tumor tissue. Recently, Milner et al. [31] in an extensive evaluation of MS110 antibody staining as a patient selection biomarker found sub-cellular localization to the nucleus. However, they concluded that although MS110 did detect BRCA1 in FFPE tumor tissue samples, BRCA1 expression levels in standard methodologies were not reproducible enough to enable its use as a selection marker.

We found less BRCA1 nuclear staining in the tumor tissue of frozen tissue specimens and in contiguous FFPE tissue with higher histological grade. The inverse correlation between lower BRCA1 protein expression and higher histological grade has been well established [24]; less nuclear staining has also been described in estrogen receptor/progesterone receptor/HER2-negative (triple negative) breast tumor tissue [32-34].

In a patient with a *BRCA1* mutation (185delAG), we found FFPE tumor tissue stained with the MS110 antibody was mostly negative and the BRCA1 staining in the FFPE normal tissue was weakly positive. These results are consistent with reports showing that heterozygous germline *BRCA1* mutations involved in familial breast cancer produce truncated protein, preferential LOH of the wild-type allele in tumor tissue, and loss of MS110 reactivity [35-37]. However, Perez-Valles et al. [29] did not find differences in nuclear BRCA1 protein expression between cases with and without BRCA1 germline mutations by immunohistochemistry.

In our study, we also found diffuse, and irregular BRCA1 protein staining with the K-18 antibody in the frozen section tumor tissue from a patient, with a *BRCA1* mutation (185delAG), which is difficult to explain. Although there is no direct evidence that the *BRCA1* 185delAG mutation results in transcription of a functional truncated protein, Buisson et al. [38] have reported that mRNAs from patients with the *BRCA1* 185delAG mutations are able to elude the non-sense mediated mRNA decay (NMD) pathway and remain stable in the cell. O’Donnell et al. [39] proposed that the truncated protein product may modulate chemo-sensitivity. Wilson et al. [24] suggest that the 185delAG mutation results in deletion of most of the protein, including the MS110 epitope which maps between aa residues 89–222. However, the 185delAG BRCA1 N-terminus 39 aa may have an epitope within aa 70–89 that is detected by the K-18 antibody.

We have found positive BRCA1 protein nuclear staining in frozen and FFPE tumor and lactating tissue from a patient with a *BRCA2* mutation. However, as our samples were tested for all three targeted mutations, there is no evidence that *BRCA1* is altered in this patient. Therefore these results are consistent with evidence that although BRCA1 protein is a negative regulator of cell growth, BRCA1 expression is up-regulated, during cell proliferation and lactation [40-42].

In our FFPE sections we frequently found that normal epithelial tissue surrounding the tumor had strong nuclear staining with the MS110 antibody, although weak staining was observed in the normal tissue in two of our specimens. This generally agrees with data that normal tissue surrounding the tumor has positive nuclear staining with the MS110 antibody [22]. However, it has also been observed by Bogdani et al. [29] using the I-20 polyclonal antibody that positive nuclear staining was found in 75% of normal tissue from both young and old patients, and that nuclear staining was absent in the normal tissue of six samples. Milner et al. [31] also found that associated non-neoplastic normal breast epithelium showed moderate to strong nuclear staining with no evidence of cytoplasmic staining in normal breast epithelium.

In summary, our immunohistological studies of frozen and FFPE tissues of breast tumors with monoclonal antibodies MS110 and AP16 and the polyclonal antibody K-18, with immunoperoxidase and immunofluorescence detection, have shown that BRCA1 protein has a nuclear and nucleolar localization. We have used freezing in liquid N2, followed by -20o C methanol fixation of the captured frozen sections in order to minimize translocation of antigen during fixation. Guerra–Rebollo et al. [43] also found that after using methanol fixation, BRCA1 was localized in nucleoli, but after ᵧ-irradiation BRCA1 was depleted from the nucleolus, and associated with ionizing-radiation induced foci (IRIF).

We have previously shown co-localization of BRCA1 protein and nucleolin in the nucleolus and speckles of MCF7 and HeLa cell nuclei [26,27]. In the present study, we found that in the mutant HCC1937 cell line (5382insC), co-localization of BRCA1 protein and nucleolin occurred in aberrant, patched regions in some nucleoli. HCC1937 extracts revealed low levels of a 210 kDa BRCA1 species consistent with the prediction of a truncated protein product lacking the BRCA1 C terminus [44]. This result suggests that protein truncation at the C-terminus may yield variable nucleolar localization. The functions of BRCA1 protein are yet to be fully elucidated, however, BRCA1 protein participates in many signaling pathways involved in transcription, checkpoint control, and is recruited for the formation of DNA repair complexes in association with proteins such as Mre11-Nbs1-Rad50, and BRCA2 [45]. The data that BRCA1 is localized in the nucleolus, in addition to speckles, may explain a possible functional participation in processes of ribosomal biosynthesis, cell cycle progression, and as a reservoir for complexes formed in response to cellular stress and DNA repair [27,43].

The function of BRCA1 protein in tumorigenesis has been found to be complex, and as a tumor suppressor, it is postulated that reduced expression leads to multiple abnormalities, including a defect in the homologous recombination (HR) pathway of DNA repair. These defects are associated with a hypersensitivity to many agents that cause DNA double strand breaks, such as ionizing radiation (IR). In a study testing breast cancer biopsies irradiated ex vivo for the ability to form BRCA1, FANCD2 and RAD 51 foci, Willers et al. [46] detected BRCA1 DNA repair foci defects in triple-negative breast cancers, a phenotype associated with BRCA1 deficiency. In addition, Burga et al. [47] found an association of increased proliferation and increased BRCA1 immunohistochemical expression in breast cancer epithelial cells from BRCA1 mutation carriers, which they ascribed to epidermal growth receptor pathway activation.

The data presented here support a role for BRCA1 in the pathogenesis of sporadic and inherited breast cancers. The use of well-characterized reagents and possibilities for co-localization experiments in both cell culture, and frozen and FFPE tissues, may lead to further insights into the molecular pathways involved in BRCA1 protein function and possibly the further development of targeted therapeutics.

## Conclusions

We compared BRCA1 protein localization in frozen and FFPE tissue, from 22 randomly selected patients with breast carcinomas. We tested 16 of the tumor specimens to determine whether they contained the common Ashkenazi Jewish founder mutations in *BRCA1* (185delAG, 5382insC), and *BRCA2* (6174delT). Two mutation carriers were identified: patient no. 4 who was a mutation carrier for *BRCA2* (6174delT), and patient no. 13 who was a mutation carrier for *BRCA1* (185delAG), and were confirmed by gene sequencing.

In the frozen sections, the BRCA1 antibody staining showed punctate, intra-nuclear granules in varying numbers of tumor, lactating, and normal cells In the FFPE tissue, we found reduced BRCA1 nuclear staining in breast tumor tissue compared to normal tissue, and less BRCA1 staining with higher histological grade in the tumors. The differences in staining between frozen and FFPE sections based on a varied group of infiltrating breast tumors, suggest that the sub-nuclear localization of BRCA1 is best studied by comparison of frozen and FFPE sections, using an array of BRCA1 antibodies, and fixation procedures.

We have previously shown co-localization of BRCA1 and nucleolin, and cell cycle dependent BRCA1 nucleolar localization in MCF7 cells. In the present study, we compared co-localization of BRCA1 and nucleolin in MCF7 cells (wild type), and a mutant *BRCA1* cell line, HCC1937 (5382insC) and found altered sub-nuclear and nucleolar localization patterns consistent with a functional impact of the mutation on protein localization.

## Materials and methods

This study was approved by the Institutional Review Board of the Mount Sinai School of Medicine. We randomly selected specimens of breast cancer tissue from 22 patients, not screened for ethnicity, which were submitted to the surgical pathology division of the Department of Pathology between 1996 and 2000 and snap froze them in liquid N2. The remainder of the tissue was routinely fixed in formalin and paraffin embedded. Family history, histopathological diagnosis, age of onset, lymph node status, and estrogen, progesterone receptor (ER, PR) status were recorded for each patient and entered into a database. The series of infiltrating breast carcinomas was classified as: ductal, lobular, medullary, colloid, or tubular; and if ductal, was graded moderately differentiated adenocarcinoma (MDA) or poorly differentiated adenocarcinoma (PDA) according to modified Bloom-Scarff-Richardson criteria [48]. Once the clinical data was collected, each patient and corresponding specimen was assigned a number; and our statistician, acting as a third-party liaison assumed responsibility for maintaining the database. DNA results were communicated to the statistician, who then entered the anonymized information into the database.

### Primary antibodies

Mouse monoclonal BRCA1 antibodies: MS110 r 1–304; MS 13 r 1–304; AP 16 r 1,313–1863; and SG 11 r-1846–1863 (22, Oncogene Research Products, CA); and the K-18 rabbit antibody, r-70–89 [49], Santa Cruz Technology, CA), were prepared at 1:20 dilution. For antigen-retrieved FFPE tissue the MS110 antibody was diluted 1:200. The specificity of the K-18 antibody binding was checked by pre-absorption with the peptide [26], Santa Cruz Technology, CA] and stained in parallel with a positive control. For nucleolin staining, mAb 7G2, a mouse monoclonal antibody was used at 1:2 [50], kind gift from S. Pinol-Roma].

### Immunohistology

The methodology for preparing the frozen sections has been described previously [26]. Briefly, tissue snap frozen in liquid N2 is mounted at about −8°C in oil (an approximately eutectic mixture of aliphatic esters with a freezing point of about −9°C), frozen at about −25°C, and sectioned using a special adhesive tape to capture the section. The section is transferred to a −13°C microscope slide coated with a UV-polymerizable adhesive and treated with a flash of UV to polymerize the adhesive and adhere the section (Leica). The slide-mounted, 6-micron frozen sections were melted at room temperature and air dried for 1 hour before being dipped for 30 to 60 sec. in −20°C methanol, and then dried for 5 minutes before being used. Before staining, sections were re-hydrated in PBS, and then incubated in primary rabbit or mouse antibodies. Normal goat serum was used to block nonspecific immunoglobulin binding and the sections were incubated in a humidified atmosphere at 4°C for 16 hours. Standard dilutions of normal rabbit or mouse IgG served as negative controls for each experiment. For immuno-peroxidase staining, the avidin-biotin-peroxidase complex technique (Dako Corp., CA) was used. For immunofluorescence, the slides were incubated with species-specific, FITC or TXR-conjugated secondary antibodies for 30 min, at a 1:100 dilution, washed extensively in PBS, and mounted with Vectashield with or without DAPI (4,6-diamidino-2-phenylindole) (Vector Labs., CA). Fluorescently stained cells or tissues were observed using a Zeiss axiophot microscope equipped with a 40X Plan-Neofluar objective, and a Leica TCS-SP (UV) confocal laser scanning microscope.

Paraffin sections were deparaffinized by heating at 60o C for 30 min followed by two 5-min immersions in xylene. The tissue sections were rehydrated through a graded series of alcohols, followed by two 15 min rinses in distilled water. They were heated in a pressure cooker for four min in 0.05 M citrate buffer (pH 5.6). Thereafter, the slides were washed twice for 15 min each in phosphate buffered saline (PBS) (Sigma). The BRCA1 monoclonal antibody MS110 produced staining of tumor cell nuclei at a 1:200 dilution. The UltraVision LP (ThermoScientific) detection system was used for diaminobenzidine (DAB) staining. Immunoreactivity was qualitatively assessed by the proportion of reactive cells and the intensity of staining: weak, faint (1), distinct (2), moderate (3), and strong (4).

### Cell culture

For the cell studies, MCF7 and HCC1937 [51] were grown in RPMI 1640 supplemented with 10% heat-inactivated FCS, 50 U/ml penicillin, and 50 μg/ml streptomycin. The cells were grown on chamber slides, washed three times in PBS, immersed in liquid N2 for 13 seconds, and then followed by 20 minute fixation in −20°C methanol. After a wash in PBS, the slides were incubated in primary rabbit or mouse antibody, or co-localized with both antibodies simultaneously. After subsequent PBS rinses, the slides were incubated with species-specific, FITC or TXR-conjugated secondary antibodies for 30 minutes, at a 1:100 dilution, washed extensively in PBS, and mounted with Vectashield with or without DAPI (Vector Labs., CA). Fluorescently stained cells were observed using a Zeiss axiophot microscope equipped with a 40x Plan-Neofluar objective, and a Leica TCS-SP (UV) confocal laser scanning microscope.

### Western blotting

For biochemical analysis, MCF7 and HCC1937 cells were grown on 100 mm culture dishes. Subconfluent, asynchronously dividing cells were rinsed once with 5 ml ice cold PBS and lysed in 1% CHAPS lysis buffer (150 mM NaCl, 10 mM HEPES, pH7.4, 1% CHAPS 3- [(3-Cholamidopropyl-dimethylammonio-1-propanesulfonate)] supplemented with protease and phosphatase inhibitors. For the detection of BRCA1, 100 μg whole cell lysate (WCL) from HCC1937 was compared to 50, 100, and 150 μg WCL from MCF7 cells and resolved using 6% SDS-PAGE. Proteins were then transferred to PVDF membrane, blocked for 1 h with 5% nonfat milk in TBS-T (20 mM Tris-CL, pH 8.0, 150 mM NaCl, 0.05% Tween 20) at room temperature, incubated with the primary antibody MS110 (AB-1) 1:500 in blocking solution overnight at 4°C, the membrane was washed three times in TBS-T, the membrane was then incubated with goat anti-mouse (HRP) secondary antibody 1: 4000 for 45 min at room temperature, washed three times 10 minutes, and proteins were detected using chemiluminescence HRP detection (Millipore).

### Genotyping

DNA from 16 anonymous breast cancer specimens was extracted from frozen tissue. A multiplex (triplex) polymerase chain reaction was performed to amplify fragments for the common Jewish Ashkenazi mutations in *BRCA1* (185delAG, 5382insC), and *BRCA2* (6174delT). The products were hybridized with normal and mutant probes for each of three mutations as previously described [12]. In accordance with IRB regulations, the anonymized DNA from the two mutation carriers was sent to Myriad Genetics (Salt Lake City, Utah) for gene sequencing. To confirm the BRCA1 185delAG mutation, DNA from FFPE slides of specimen no.13 was isolated using the BiOstic FFPE tissue DNA isolation kit (Mo Bio Laboratories) and amplified by PCR using the oligonucleotide primers flanking the mutation loci: BRCA1, 185delAG forward (5′-GGATTTATCTGCTCTTCGCGTT-3′) and 185delAG reverse (5′-TGTCTTTTCTTCCCTAGTATGT-3′). The PCR product was purified and subjected to DNA sequencing with the primers used for the amplification. As a negative control, DNA from specimen no.12 was amplified and sequenced using the same primers. The sequences were compared with the reference sequences using program BLAST.

## Competing interests

The authors declare they have no competing interests.

## Authors’ contributions

NT conceived the study, LO helped to analyze the data, SD, JS, IB, SJ, CN provided samples, histopathological diagnoses, and analyzed data, RK, LE, KB did screening, and analysis for 3 common Ashkenazi mutations BRCA1 (185delAG, 5382insC) and BRCA2 (6174delT), CB maintained the database and collated the genetic data, VDN did molecular genetic studies on FFPE tissue, MC and ANAM critically revised the manuscript, NTW did the western blotting. All authors read and approved the final manuscript.
